# Effects of dynamic [^18^F]NaF PET scan duration on kinetic uptake parameters in the knee

**DOI:** 10.3389/fnume.2023.1194961

**Published:** 2023-11-24

**Authors:** Lauren E. Watkins, Bryan Haddock, Ananya Goyal, Feliks Kogan

**Affiliations:** ^1^ Department of Radiology, Stanford University, Stanford, CA, United States; ^2^ Department of Imaging Research, Steadman Philippon Research Institute, Vail, CO, United States; ^3^ Department of Clinical Physiology, Nuclear Medicine and PET, Rigshospitalet, Copenhagen University Hospital, Copenhagen, Denmark; ^4^ Department of Bioengineering, Stanford University, Stanford, CA, United States

**Keywords:** [^18^F]NaF PET, scan duration, Hawkins model, osteoarthritis, kinetic uptake parameters

## Abstract

**Introduction:**

Accurately estimating bone perfusion and metabolism using [^18^F]NaF kinetics from shorter scan times could help address concerns related to patient comfort, motion, and throughput for PET scans. We examined the impact of changing the PET scan duration on the accuracy of [^18^F]NaF kinetic parameters in the knee.

**Methods:**

Both knees of twenty participants with and without osteoarthritis were scanned using a hybrid PET-MRI system (53 ± 13 years, BMI 25.9 ± 4.2 kg/m^2^, 13 female). Seventeen participants were scanned for 54 ± 2 min, and an additional three participants were scanned for 75 min. Patlak *K*_i_ and Hawkins kinetic parameters (*K*_i_, *K*_1_, extraction fraction) were assessed using 50- or 75-minutes of scan data as well as for scan durations that were retrospectively shortened. The error of the kinetic uptake parameters was calculated in bone regions throughout the knee.

**Results:**

The mean error of Patlak *K*_i_, Hawkins *K*_i_, *K*_1_, and extraction fraction was less than 10% for scan durations exceeding 30 min and decreased with increasing scan duration.

**Conclusions:**

The length of dynamic data acquisition can be reduced to as short as 30 min while retaining accuracy within the limits of reproducibility of Hawkins kinetic uptake parameters.

## 1. Introduction

[^18^F]sodium fluoride [(^18^F)NaF] positron emission tomography (PET) imaging is a promising technique to study the role of bone metabolism in joint diseases such as osteoarthritis (1). [^18^F]NaF uptake is influenced by regional bone blood flow, or perfusion, and bone metabolism. Tracer uptake kinetics may be quantified using dynamic scans of up to 60 min or longer. The rate of fluoride clearance from plasma to the bone matrix (*K*_i_) can be estimated using the graphical Patlak model (2) or using nonlinear regression based on the Hawkins two-tissue model (3). The Hawkins method can further be used to estimate bone perfusion (*K*_1_) as well as the fraction of [^18^F] extracted into the bone where it binds to the bone matrix in newly formed hydroxyapatite crystals at sites of bone formation (4) [extraction fraction, or *k*_3_/(*k*_2 _+ *k*_3_)]. The Hawkins *K*_i_ has good reproducibility, with a precision error of less than 15% when calculated from 50 to 60 min of dynamic PET data (5–8). However, long scan times can result in increased motion artifacts, reduced throughput, and patient discomfort (9). Methods of accurately estimating [^18^F]NaF uptake kinetics from shorter acquisitions could help address these concerns.

Here, we examined the impact of shortening dynamic PET acquisition duration on kinetic [^18^F]NaF uptake parameters in the knee, hypothesizing that the scan duration could be shortened without impacting the accuracy of kinetic uptake parameters.

## 2. Methods

### 2.1. Study population

This study was approved by the University Institutional Review Board and all participants signed an informed consent form. Participants included 20 volunteers with (*n* = 12) and without (*n* = 8) clinically diagnosed knee osteoarthritis (53 ± 13 years, BMI 25.9 ± 4.2 kg/m^2^, 13 female).

### 2.2. Image acquisition

Both knees of all participants were scanned simultaneously using a 3 T whole-body time-of-flight hybrid PET-MRI system (GE SIGNA, GE Healthcare, Milwaukee WI) with two 16-channel flexible phased-array wrap coils. Both knees were scanned in one PET bed position in list mode [field of view: 25 cm axial × 60 cm trans-axial, full width at half maximum at 1 cm axial: 4.66 mm (10)] immediately following intravenous injection of 91.2 ± 6.6 MBq (0.8 ± 0.4 ml) of [^18^F]NaF (SOFIE Biosciences, Gilroy CA). Seventeen participants were scanned for 50–60 min (54 ± 2 min), and an additional 3 healthy participants were scanned for 75 min.

### 2.3. Image reconstruction

**Figure 1:**
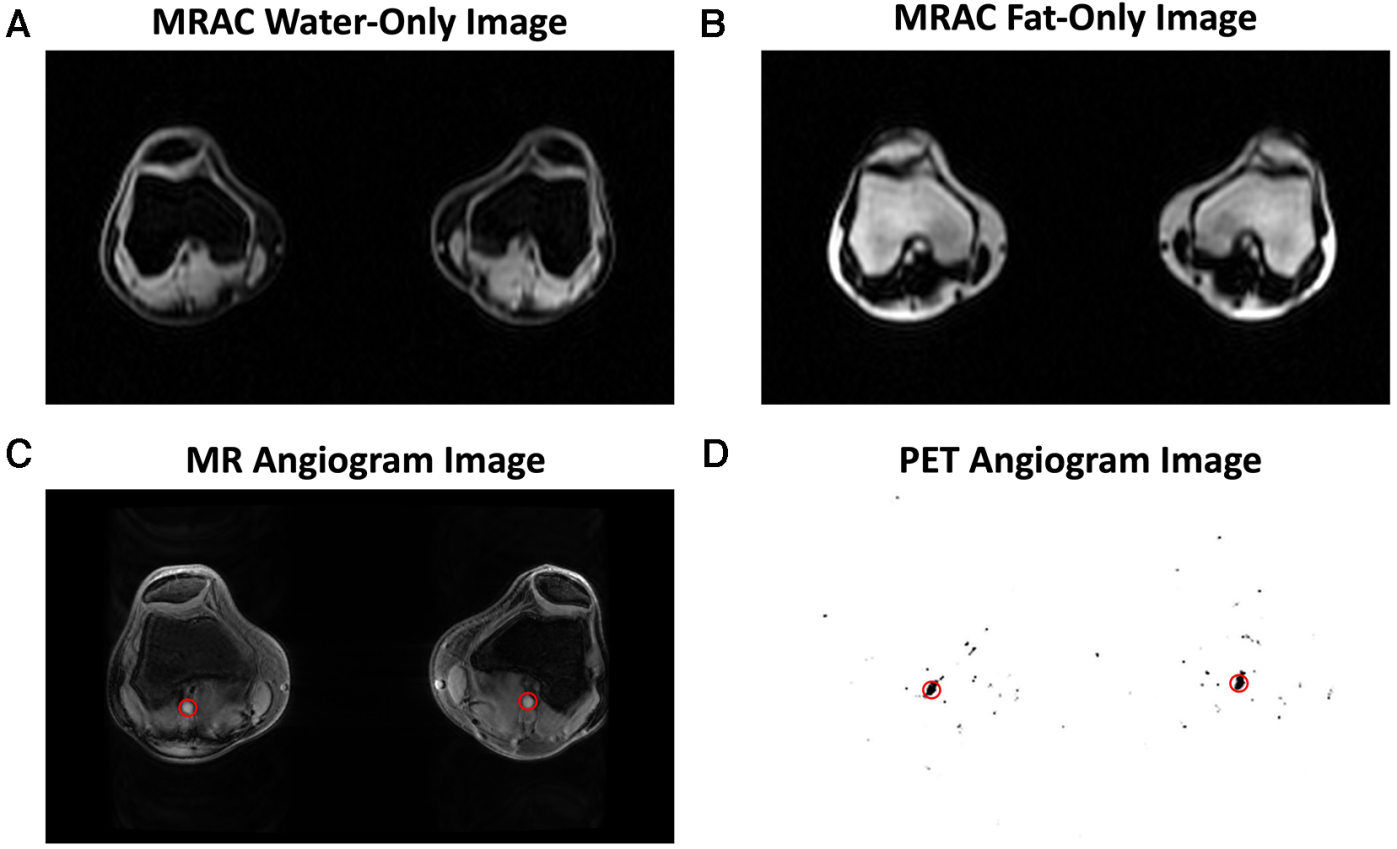
Representative images are shown for the magnetic resonance imaging (MRI) and positron emission tomography (PET) images used in image analysis. MR-based attenuation correction (MRAC) of PET data was performed using a 2-point Dixon fat-water T1-weighted spoiled gradient echo MR sequence, which generated a water-only image (**A**) and fat-only image (**B**). An MR angiogram (**C**) and PET angiogram (**D**) were used to automatically segment the popliteal arteries (red circles) and derive the arterial input function.

**Figure 2:**
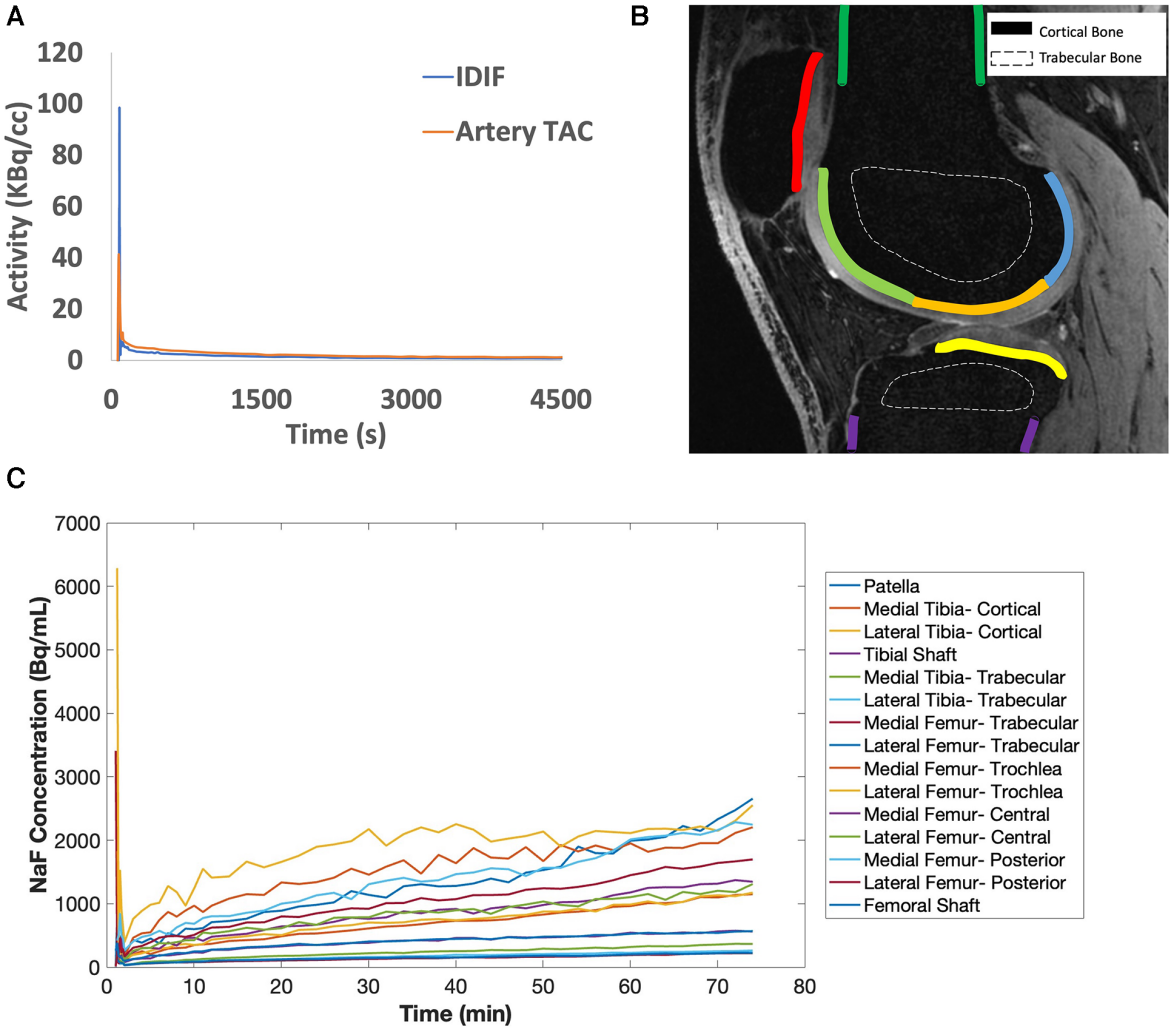
(**A**) [^18^F]sodium fluoride [(^18^F)NaF] uptake in the popliteal arteries was assessed using an image-derived input function (IDIF, blue line), which has a similar shape as the time activity curve (TAC) in the popliteal artery (artery TAC, orange line). (**B**) Bone was segmented from quantitative double-echo in steady state (qDESS) images and refined via a custom MATLAB script to derive a total of 15 bone subregions for cortical (solid lines) and trabecular bone (dashed lines). In the qDESS image shown: dark green = cortical bone of the femoral shaft, light green = subchondral bone of the trochlear region of the femur, orange = subchondral bone of the central region of the femur, blue = subchondral bone for the posterior region of the femur, yellow = subchondral bone for the tibia, purple = cortical bone of the tibial shaft, and red = subchondral bone for the patella. (**C**) Example tissue TACs are shown for each of the 15 bone subregions per knee.

MRI was performed simultaneously with PET acquisition. MR-based attenuation correction of PET data was performed using a 2-point Dixon fat-water T1-weighted spoiled gradient echo MR sequence (11, 12) with TR/TE1/TE2 = 4.1/1.1/2.2 ms, FOV = 50 × 37.5 cm, matrix = 256 × 128, slice thickness = 5.2 mm (Figures 1A,B). MR angiography images, for segmentation and measurement of the popliteal artery volume, were acquired using a 3D inversion recovery spoiled gradient echo (IR-SPGR) sequence with repetition time (TR)/echo time (TE) = 21/2.1 ms, matrix = 512 × 512, number of slices = 18, slice thickness = 1.2 mm (Figure 1C). Quantitative double-echo in steady state (qDESS) images acquired for tissue segmentation had TR/TE1/TE2 = 24.6/5.8/43.4 ms, FOV = 16 cm, matrix = 320 × 320, and slice thickness = 1.5 mm (Figure 2B, Figure 4).

All PET image frames were reconstructed from acquired list-mode data using a time-of-flight reconstruction (∼400 picosecond timing resolution) with resolution recovery corrections and a regularized reconstruction iterative algorithm [QClear, beta value of 350 (13)]. Reconstructed image frames had a matrix size of 384 × 384 and voxel size of 1.30 mm × 1.30 mm × 2.78 mm. An image-derived input function (IDIF) was determined from [^18^F]NaF activity (kBq/ml) in the popliteal artery of each knee independently, as previously described (1, 14, 15). With this technique, differences between venous blood samples and IDIF values measured at 50 min were on average within 0.2 kBq/ml, which corresponds to a coefficient of variation of 12% (Supplementary Figure S1). Dynamic PET frames were reconstructed with frame times: 20 × 1 s, 10 × 10 s, 10 × 30 s, 5 × 1 min, and 2 min frames for the remaining duration of the scan to calculate the IDIF (Figure 2A). First, the popliteal arteries were automatically segmented from MR angiography images and a short time-frame PET angiogram (0–16 s after injection) (14). Representative images are shown in Figures 1C,D, where the popliteal artery diameter was 5.5 (right) and 6.0 mm (left), in agreement with published ranges for artery diameters in adults (16, 17). To minimize spill-over artifacts (18), the voxels centered in the middle of the artery were determined for each dynamic frame and used for the IDIF region of interest. Centered voxels were defined by including the voxels in each axial slice within the highest 10% of arterial [^18^F]NaF activity and as having a positive gradient in the three dynamic frames preceding the bolus peak (3 s) and a negative post-peak gradient. A 3-ml venous blood sample was taken at the end of the scan (50 or 75 min after injection) and measured in a well counter to confirm that IDIF activity measures were similar to venous blood sample measurements. The venous sample was not utilized to calibrate the input function nor was a population-based input function utilized. Further, a time activity curve (TAC) of bone tracer uptake was reconstructed from dynamic list mode PET data with frame times: 6 × 10 s, 10 × 1 min, and 2 min frames for the remaining duration of the scan.

### 2.4. Segmentation

Tracer uptake was calculated in regions in the patella, femur, and tibia representing cortical, subchondral, and trabecular bone as described previously (14). Briefly, qDESS images were first re-sampled to PET resolution using Horos software and used to manually create masks of the entire femur, patella, and tibia in ITK-Snap (19). Bone masks were then segmented further to separate trabecular, subchondral, and cortical bone masks using *k*-means clustering of registered water-only and fat-only Dixon images (14). Cortical bone at the patellar tendon epiphyses was excluded. Subchondral bone masks were manually subdivided into regions representing the patella as well as the medial and lateral tibia and femur, then each portion of the femur was further manually subdivided into trochlear, central, and posterior regions. There were 15 bone regions per knee (Figure 2B, representative TAC curve for each bone region in Figure 2C).

### 2.5. Kinetic uptake parameters

**Figure 3:**
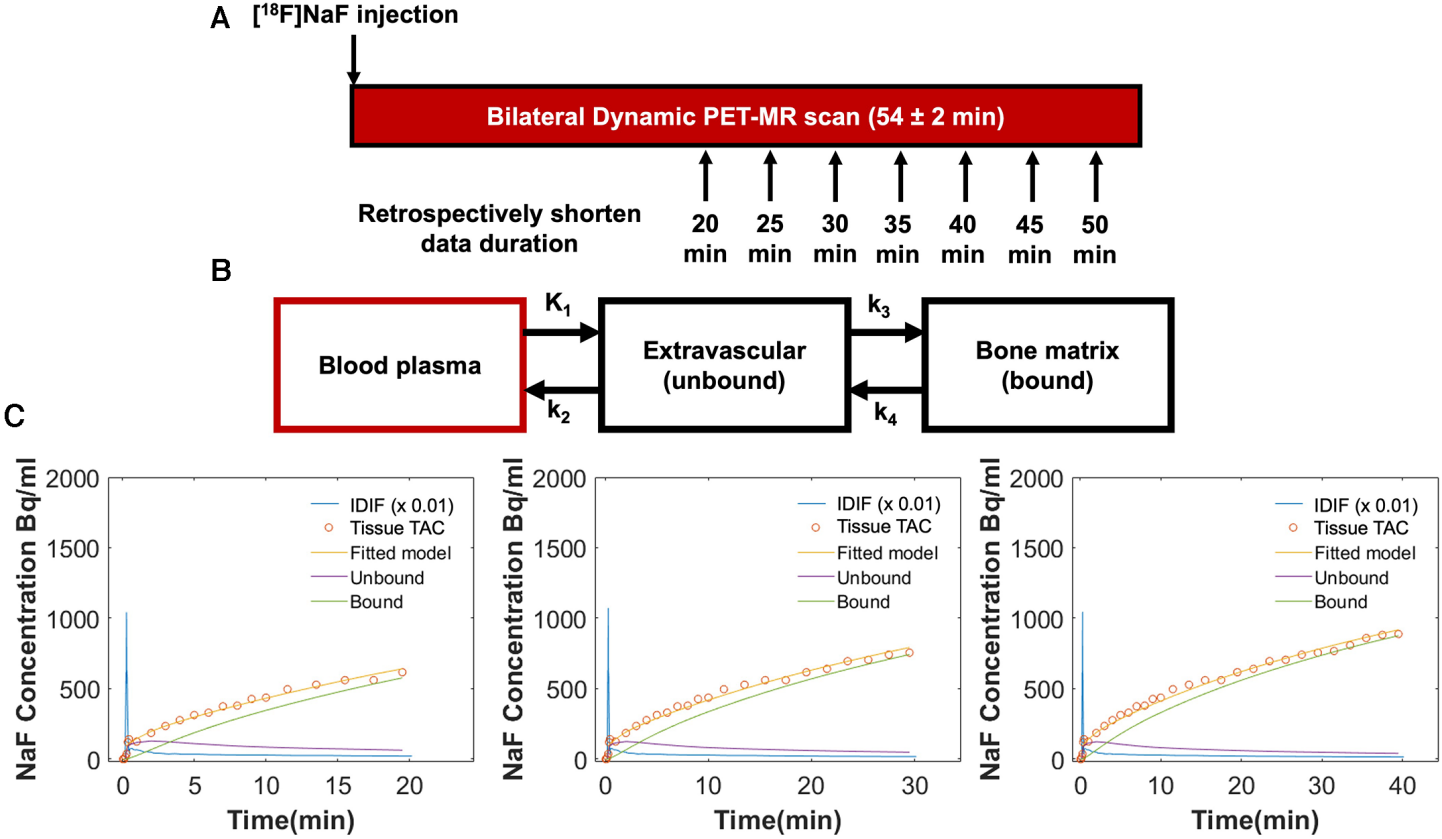
(**A**) Both knees of all participants were scanned simultaneously using a 3 T hybrid PET-MRI system immediately following intravenous injection of [^18^F]sodium fluoride [(^18^F)NaF]. Seventeen participants were scanned for a minimum of 50 min (54 ± 2 min), and an additional three healthy participants were scanned for 75 min. Tracer uptake data was retrospectively shortened to 20, 25, 30, 35, 40, and 45 min. (**B**) Tracer uptake data (IDIF and tissue TAC) for each duration was fit to a Hawkins two-tissue tracer kinetic model to estimate rate constants for individual bone regions: *K*_1_ = bone perfusion (ml min^−1^ ml^−1^), *k*_2_ = tissue clearance (min^−1^), and *k*_3_ = mineralization (min^−1^). *K*_4_ was set to 0. From these rate constants, extraction fraction [the fraction of (^18^F) entering the bone tissue that binds to the bone matrix] was defined as *k*_3_/[*k*_2 _+ *k*_3_] and *K*_i_ [the rate of clearance of (^18^F) from the plasma to the bone mineral compartment] was calculated as *K*_1_*extraction fraction (ml min^−1^ ml^−1^). (**C**) Representative Hawkins model fits are shown for data of 20, 30, and 40 min of duration for the same bone region in an osteoarthritic knee. At 20 min, *K*_i_ = 0.0121 ml min^−1 ^ml^−1^, *K*_1_ = 0.015 ml min^−1 ^ml^−1^, and extraction fraction = 0.81; at 30 min, *K*_i_ = 0.0116 ml min^−1 ^ml^−1^, *K*_1_ = 0.015 ml min^−1 ^ml^−1^, and extraction fraction = 0.78; at 40 min, *K*_i_ = 0.0096 ml min^−1 ^ml^−1^, *K*_1_ = 0.013 ml min^−1 ^ml^−1^, and extraction fraction = 0.75.

Kinetic rate constants: *K*_1 _= bone perfusion (ml min^−1^ ml^−1^), *k*_2_ = tissue clearance (min^−1^), *k*_3_ = mineralization (min^−1^) and metabolism (Ki, ml min^−1^ ml^−1^) were calculated for individual bone regions as described above. For kinetic modeling of tracer uptake, the IDIF and tissue TAC data were fit to a Hawkins two-tissue tracer kinetic model (Figure 3) using a nonlinear regression (NLR) method as described previously (3). Given the signal to noise in the knee, the small value of the rate constant *k*_4_ (0.01/min), and the shorter scan durations considered in this study, *k*_4_ was defined as 0 (8). NLR fitting to estimate these three rate parameters, along with parameters to account for partial volume fraction, blood fraction, and input dispersion estimate (a total of 6 parameters) was performed for each bone region using COMKAT software (20). Extraction fraction was defined as *k*_3_/[*k*_2 _+ *k*_3_] and represents the fraction of [^18^F] entering the bone tissue that binds to the bone matrix (as opposed to being cleared back into the plasma pool) and ranges in value from 0 to 1. *K*_i_^NLR^ (*K*_i_), the rate of clearance of [^18^F] from the plasma to the bone mineral compartment, was calculated as *K*_1_*extraction fraction with units of mL min^−1^ ml^−1^. For comparison to previous literature examining uptake parameter estimation with shortened protocols (21, 22), *K*_i_ was also calculated using the graphical Patlak method (2) using uptake data starting at 10 min for fitting to ignore the non-linear part of the curve during the non-equilibrium state of the system (23).

### 2.6. Data analysis

Kinetic parameters were first calculated using 50 min of scan data for all participants. To examine how abbreviated PET scan times might impact kinetic uptake parameters, Patlak and Hawkins kinetic modeling was also performed for retrospectively shortened IDIF and tissue TAC data of only 20-, 25-, 30-, 35-, 40-, or 45-minutes duration (Figure 3). For the 3 participants scanned for 75 min, the same methods were repeated comparing the kinetic parameters obtained from the 75 min of data to those obtained from shortened data sets. The mean and standard deviation of the normalized difference (error, %) of the kinetic uptake parameters derived from the shortened scan data were calculated with reference to parameters derived from modeling the original scan data. Finally, since signal to noise is important to consider, error was reported separately for bone regions with relatively “high” uptake (*K*_i_ > 0.03 ml min^−1^ ml^−1^), “low” uptake (*K*_i_ < 0.01 ml min^−1^ ml^−1^), and “medium” uptake (*K*_i_ between 0.01–0.03 ml min^−1^ ml^−1^).

## 3. Results

Across all bone regions and participants, the mean error of the Hawkins *K*_i_, *K*_1_, and extraction fraction, and the Patlak *K*_i_, was less than 6% for all scan durations with respect to parameters calculated using 50 min of scan data. Representative Hawkins model fits used to estimate kinetic parameters for shortened scan durations are shown in Figure 3C. A summary of the volumes of each bone region and y-axis intercept values from Patlak analysis (V_0_) are provided in the Supplemental Material. Mean and standard deviation in values and percent errors for Patlak *K*_i,_ Hawkins *K*_i_, *K*_1_, and extraction fraction are presented in Tables 1, 2 respectively. The standard deviation of the error was 15% or less for scan durations of 30 min or longer.

**Table 1:** Mean and standard deviation in the kinetic parameters for each scan duration.

Scan duration (min)	Patlak *K*_i_	Hawkins *K*_i_	*K*_1_	Extraction fraction
	(ml min^−1^ ml^−1^)	(ml min^−1^ ml^−1^)	(ml min^−1^ ml^−1^)	
20	0.0085 ± 0.0087	0.0101 ± 0.0096	0.0172 ± 0.0169	0.669 ± 0.231
25	0.0085 ± 0.0087	0.0101 ± 0.0096	0.0171 ± 0.0170	0.677 ± 0.220
30	0.0083 ± 0.0083	0.0100 ± 0.0088	0.0172 ± 0.0170	0.678 ± 0.210
35	0.0082 ± 0.0081	0.0098 ± 0.0086	0.0170 ± 0.0168	0.677 ± 0.211
40	0.0081 ± 0.0079	0.0096 ± 0.0084	0.0169 ± 0.0167	0.674 ± 0.213
45	0.0080 ± 0.0078	0.0095 ± 0.0083	0.0168 ± 0.0168	0.673 ± 0.213
50	0.0085 ± 0.0074	0.0102 ± 0.0078	0.0177 ± 0.0162	0.675 ± 0.213

**Table 2:** Mean and standard deviation of the percent error of kinetic uptake parameters from shortened scan data compared to 50-minute scan data.

Scan duration (min)	Patlak *K*_i_	Hawkins *K*_i_	*K*_1_	Extraction fraction
20	5.17 ± 16.02	3.38 ± 25.07	4.31 ± 19.53	0.89 ± 25.70
25	5.09 ± 12.98	4.00 ± 21.52	2.85 ± 16.70	2.22 ± 21.16
30	4.09 ± 9.61	5.12 ± 14.93	3.21 ± 13.58	2.70 ± 15.37
35	2.56 ± 7.78	3.35 ± 13.08	2.34 ± 12.52	1.65 ± 12.72
40	1.58 ± 5.09	1.87 ± 10.15	1.46 ± 11.33	0.88 ± 9.46
45	0.71 ± 3.24	0.60 ± 7.68	0.38 ± 8.67	0.46 ± 6.09

**Figure 4:**
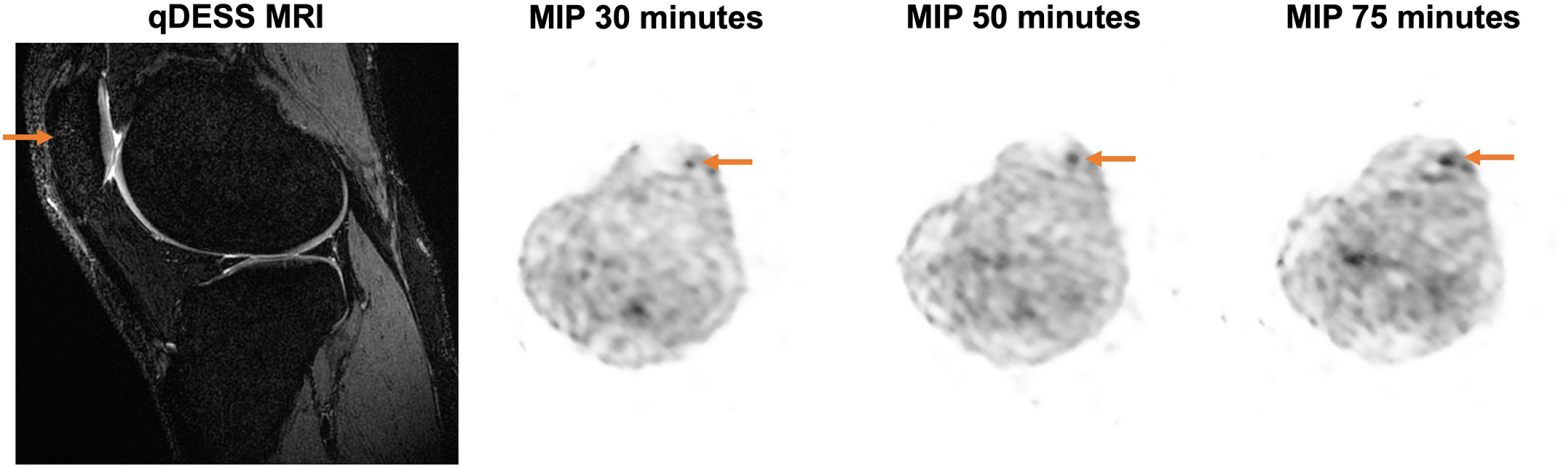
Representative MRI and maximum intensity projection (MIP) PET images are shown for a 27-year-old female healthy participant with a BMI of 19.94 kg/m^2^. A sagittal qDESS MRI shows a structurally unremarkable patella; however, MIP PET images demonstrate an area of focally high uptake (arrow) at the 30-, 50-, and 75-minute timepoints.

An example qDESS MRI and maximum intensity projection (MIP) PET images of tracer uptake at 30, 50, and 75 min depict an area of focally high tracer uptake in the patella at each timepoint for a healthy knee (Figure 4). For the three healthy subjects scanned for 75 min, the mean and standard deviation of the kinetic uptake parameters at 75 min were 0.0054 ± 0.0027 ml min^−1 ^ml^−1^ for Patlak *K*_i_, 0.0067 ± 0.0032 ml min^−1 ^ml^−1^ for Hawkins *K*_i_, 0.0095 ± 0.0048 for *K*_1_ ml min^−1^ ml^−1^, and 0.745 ± 0.180 for extraction fraction. Errors in the estimation of kinetic parameters associated with using shortened scan durations compared to parameters calculated using 75 min of scan data as a reference are presented in Table 3. Negative values represent underestimations of uptake parameters relative to those calculated using 75 min of scan data. For scan durations 30 min or longer, the mean and standard deviation of the error was less than 15% for all parameters. The errors decreased with increasing scan duration.

**Table 3:** Mean and standard deviation of the percent error of kinetic uptake parameters from shortened scan data compared to 75-minute scan data.

Scan duration (min)	Patlak *K*_i_	Hawkins *K*_i_	*K*_1_	Extraction fraction
20	−6.59 ± 18.55	−5.95 ± 22.50	4.24 ± 23.39	−7.10 ± 26.64
25	−5.54 ± 14.77	−9.51 ± 18.27	4.27 ± 20.54	−11.75 ± 18.32
30	−3.06 ± 13.65	−4.51 ± 14.65	1.23 ± 14.97	−4.89 ± 13.63
35	−2.28 ± 12.42	−4.80 ± 13.71	1.38 ± 13.52	−5.55 ± 11.95
40	−1.38 ± 11.55	−3.31 ± 12.34	0.07 ± 12.92	−2.82 ± 10.77
45	−1.77 ± 10.99	−2.43 ± 13.17	0.11 ± 11.58	−2.07 ± 12.45
50	−2.35 ± 10.91	3.45 ± 14.06	−1.36 ± 12.27	−1.39 ± 14.96
55	−2.53 ± 9.06	−3.08 ± 11.89	−0.09 ± 10.82	−2.65 ± 10.55
60	−1.75 ± 5.95	−1.27 ± 9.60	−0.32 ± 11.02	−0.34 ± 9.74
65	−1.02 ± 3.89	0.16 ± 9.31	1.22 ± 9.97	−0.83 ± 6.06
70	−0.35 ± 1.69	−0.11 ± 7.58	−0.76 ± 9.54	1.11 ± 7.72

**Figure 5:**
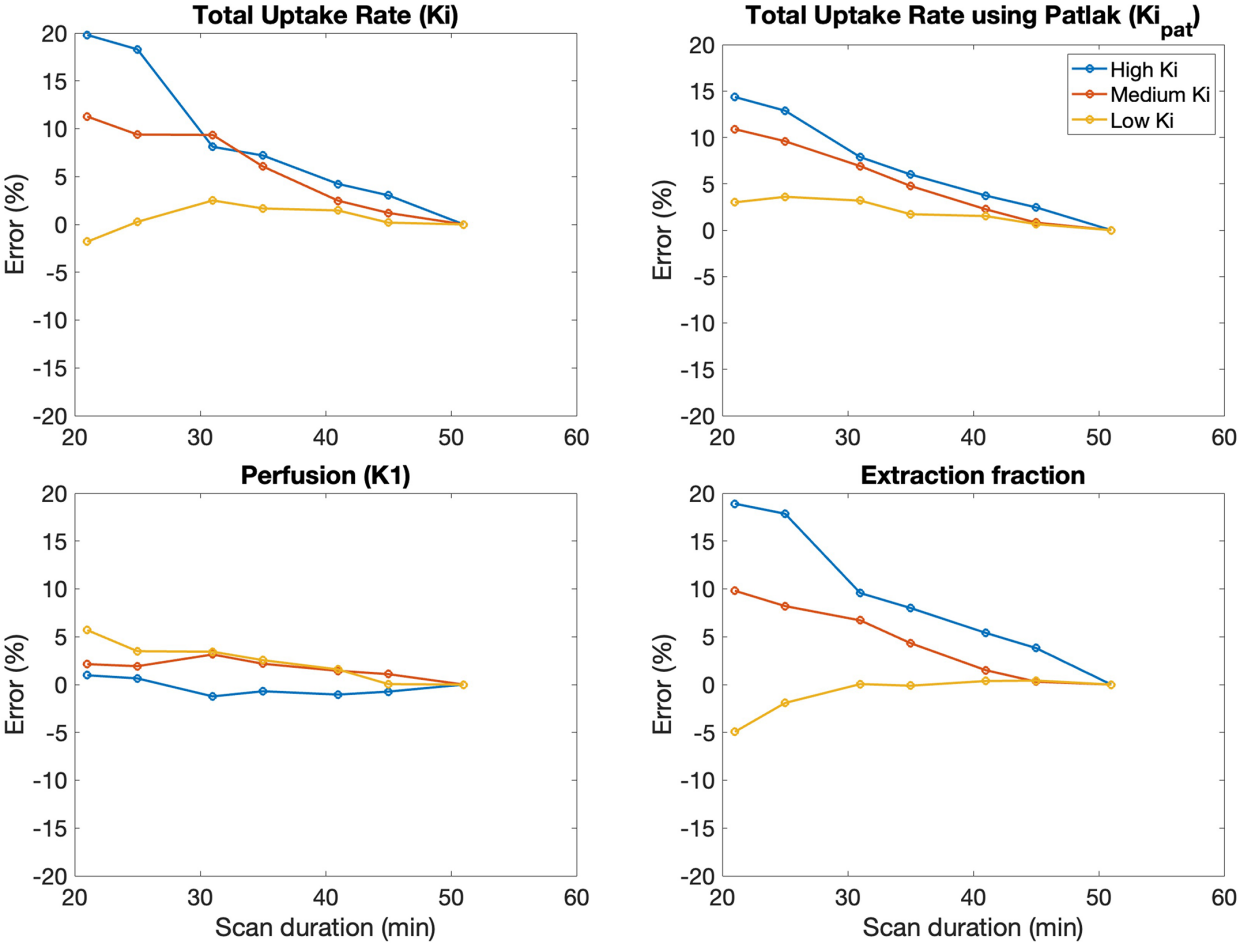
The average error in kinetic uptake parameters associated with shortened scan durations was reported separately for bone regions with relatively “high” uptake (*K*_i _> 0.03 ml min^−1 ^ml^−1^), “medium” uptake (*K*_i_ between 0.01–0.03 ml min^−1 ^ml^−1^), and “low” uptake (*K*_i _< 0.01 ml min^−1 ^ml^−1^). Error was calculated with reference to the parameters derived from a 50-minute scan. For all kinetic uptake parameters, error decreased with increasing duration of the scan data used for fitting the models.

Error in kinetic parameters grouped by [^18^F]NaF uptake rates is shown in Figure 5 and Table 4. There were 14 regions with high *K*_i_, 144 regions with medium *K*_i_, and 442 regions with low *K*_i_. The mean error at 50 min is 0% since parameters derived from a 50-minute scan were used as a reference. The error decreased with increasing duration of the scan data used for fitting the models, and was less than 10% for high, medium, and low uptake rates when calculated using data with a duration of at least 30 min.

**Table 4:** Mean and standard deviation of the percent error in kinetic uptake parameters in bone regions with low (*K*_i_ < 0.01 ml min^−1 ^ml^−1^), medium (*K*_i_ between 0.01–0.03 ml min^−1 ^ml^−1^), and high (*K*_i_ > 0.03 ml min^−1 ^ml^−1^) *K*_i_ values.

Scan duration (min)	Patlak *K*_i_			Hawkins *K*_i_		
	High *K*_i_	Medium *K*_i_	Low *K*_i_	High *K*_i_	Medium *K*_i_	Low *K*_i_
20	14.35 ± 13.32	10.89 ± 15.92	3.00 ± 15.25	19.80 ± 16.61	11.28 ± 24.05	−1.81 ± 24.50
25	12.88 ± 12.47	9.59 ± 12.45	3.60 ± 12.85	18.30 ± 18.27	9.39 ± 18.16	0.27 ± 22.57
30	7.87 ± 8.41	6.90 ± 8.92	3.18 ± 9.49	8.11 ± 9.62	9.35 ± 13.64	2.50 ± 15.26
35	6.01 ± 5.65	4.77 ± 7.23	1.72 ± 7.54	7.20 ± 7.09	6.05 ± 11.55	1.67 ± 13.79
40	3.73 ± 3.24	2.25 ± 4.09	1.52 ± 5.35	4.23 ± 3.56	2.48 ± 8.58	1.46 ± 11.04
45	2.46 ± 2.02	0.88 ± 2.52	0.65 ± 3.44	3.04 ± 4.5	1.20 ± 6.91	0.18 ± 8.14
50	0	0	0	0	0	0
Scan duration (min)	*K*_1_			Extraction fraction		
	High *K*_i_	Medium *K*_i_	Low *K*_i_	High *K*_i_	Medium *K*_i_	Low *K*_i_
20	−0.97 ± 5.86	2.13 ± 13.74	5.69 ± 22.40	18.91 ± 17.02	9.81 ± 23.98	−4.94 ± 25.18
25	−0.65 ± 5.01	1.92 ± 14.10	3.49 ± 18.33	17.86 ± 19.06	8.20 ± 18.36	−1.92 ± 21.69
30	−1.24 ± 4.20	3.15 ± 12.38	3.43 ± 14.46	9.46 ± 9.80	6.71 ± 13.98	0.05 ± 15.77
35	−0.70 ± 3.46	2.18 ± 11.18	2.55 ± 13.43	8.00 ± 6.89	4.33 ± 11.26	−0.12 ± 13.34
40	−1.05 ± 2.17	1.44 ± 11.57	1.58 ± 11.42	5.40 ± 4.75	1.50 ± 7.60	0.37 ± 10.38
45	−0.74 ± 2.49	1.09 ± 8.28	0.05 ± 9.01	3.82 ± 4.10	0.30 ± 4.70	0.42 ± 6.76
50	0	0	0	0	0	0

## 4. Discussion

We examined the impact of shorter scan durations on estimating kinetic parameters, including Hawkins parameters for bone perfusion (*K*_1_), extraction fraction [*k*_3_/(*k*_2 _+ *k*_3_)], and metabolism (*K*_i_) from [^18^F]NaF uptake. Retrospectively shortening the duration of the time activity curve increased errors in *K*_i_, *K*_1_, and extraction fraction. Trends were similar between Hawkins and Patlak techniques, though the Patlak *K*_i_ was associated with lower variability and the parameter V_0_ from the graphical Patlak analysis increased in a linear fashion as scan time decreased. The mean error in Hawkins uptake parameters were, however, less than 10% for scan durations exceeding 30 min for regions with high and low uptake. Errors in kinetic uptake parameters were comparable between Hawkins and Patlak models.

Conventional techniques for kinetic quantification using [^18^F]NaF require data from a 60-minute dynamic scan (23). Precision error using the 60-minute scans are reported to range from 13.9–14.5% for Hawkins *K*_i_ and 11.7–13.5% for Patlak *K*_i_ (6, 7). The precision error metric from reproducibility studies, defined as the percentage of the standard deviation divided by the mean from two repeated PET scans (6), is comparable to the standard deviation of the percent error between abbreviated and full-length scans used in this study. For scan durations of 30 min or longer, the standard deviations in the error were within previously reported ranges for precision error at 60 min (23) for all kinetic uptake parameters. Further, the errors in parametric values with shortened scan durations are similar to prior literature on new techniques for quantifying [^18^F]NaF uptake rates from shorter acquisitions (22), including from a 12-minute dynamic scan using a Hawkins model with fixed rate constants (24). With the 12-minute technique, the Hawkins *K*_i_ had an equivalent or superior statistical power compared to the conventional scan in an osteoporotic population. However, with fixed rate constants, *K*_1_ values were in a fixed ratio to *K*_i_ and did not represent true measurements of bone blood flow. In the current study, both *K*_i_ and associated Hawkins rate constants were freely fitted to available scan data, which may be more widely applicable to a variety of conditions where bone metabolism may vary.

Although studies typically use scan times of around an hour, increased scanning time, such as in the 75-minute datasets in the present study, give similar results. Kinetic uptake parameters for all abbreviated scan durations were within 5% of values calculated from 50 min of scan data and within 12% of values calculated from 75 min of scan data; for both, errors were within 5% for scan durations exceeding 30 min. PET images show similar patterns of tracer uptake at 30, 50, and 75 min. At 30 min, it can be assumed that circulating venous and arterial [^18^F]NaF have reached equilibrium (25). A 30-minute scan may thus represent a reasonable lower limit for abbreviated dynamic acquisitions without appreciable impacts on parametric values. Introducing errors on the order of 5% from shorter acquisitions may be an acceptable compromise to reduce overall study time and motion artifacts for exercise studies, for example, where relative changes in kinetic uptake parameters on the order of 25%–180% have been observed (26).

SUV, *K*_i_, and *K*_1_ have been observed to be elevated in osteoarthritic knees compared to healthy ones (1), although areas of high uptake may occur in non-osteoarthritic knees (27) and after exercise in healthy knees (26). To account for the variable uptake levels that may be found within a given knee or in situations like loading that induce high tracer uptake, differences in errors at each scan duration were examined according to tracer uptake levels. The overall error in uptake parameters decreased with increasing scan durations but was variable between bone regions with high or low uptake rates. Bone regions with moderate to high uptake rates had higher SNR contributing to lower error in *K*_1_ with shorter scan durations but larger errors in the extraction fraction. Extraction fraction is generally associated with greater variability than *K*_1_ due to greater uncertainties in estimating rate constants *k*_2_ and *k*_3_ (28). Additionally, extraction fraction in bone regions with moderate-high uptake is more variable than in bone regions with low uptake, as observed in prior work in an osteoarthritic population and exercise studies where high uptake rates were associated with [^18^F] delivery that outpaced clearance (1, 26, 29). Errors in kinetic parameters calculated with reference to a 75-minute PET acquisition followed similar trends. Shortening the scan duration to 30 min was sufficient to estimate kinetic parameters within 10% of parameters calculated using 50 and 75 min of scan data. Using shorter scans with fewer time points or shorter frames does change the calculus regarding signal to noise. Although regions of interest within the knee are small curved shapes where smaller voxels are advantageous for segmentation and contrast recovery (30, 31), the combined signal to noise of the volume does need to be sufficient for fitting parameters requiring an optimization of frame time, number of frames and voxel size.

One limitation of this work was the variability in the number of regions with different *K*_i_ values in this population including osteoarthritic knees. The selection of *K*_i_ ranges was based on prior work in an osteoarthritic population (1), but there were fewer bone regions with high *K*_i_ compared to regions with low *K*_i_, which may have impacted the observed differences in mean error between groups. While it is a heterogeneous sample, the division of results by bone regions with different uptake rates attempted to represent regional and metabolic variability associated with disease and associated impacts on errors observed in shortened scans. Regions of low and high bone metabolism can be found in knees with conditions affecting bone metabolism such as osteoarthritis, so it is important to consider this variability when using abbreviated protocols. Another limitation was that only three datasets with 75 min of data were used to compare errors against datasets with 50–60 min of data. This data was used as a proof of concept to demonstrate that results were not noticeably different when longer duration scans were used as a reference. Additionally, k-loss due to *k*_4_ was not assessed for longer scan times. As assessed by Siddique et al., k-loss due to *k*_4_ can introduce errors underestimating *K*_i_ by approximately 20% after 2 h (32). However, in this dataset only three participants were scanned for longer than 50 min and, for these three datasets, the scan time was only 75 min which is a short time frame compared to the 120 min used previously to determine *K*_i_ loss. Moreover, we aimed to focus our analysis on the shorter scan durations, where *k*_4_ is small (∼0.01/min) and is commonly considered negligible. Bone was not considered in the attenuation correction technique and while this may affect the accuracy of quantifying tracer uptake, this would have a similar impact at all durations studied. The same image-derived input function was used for the arterial input function (14, 15) for all retrospectively time-under sampled pharmacokinetic model fitting. Multiple plasma blood measurements were not used for validation; however, this technique has previously shown good reproducibility (14, 15). Minor discrepancies observed between the IDIF and artery TAC following peak tracer delivery may be attributed to a known bias where the IDIF underestimates measures on the order of 2%–5% (15) or may possibly reflect tracer uptake in arterial walls. Alternative input functions were beyond the scope of this work and were not explored. Finally, a limitation of techniques incorporating shorter acquisitions is that SUV will be lower with shorter uptake times. SUV could still be incorporated in population studies with shorter protocols as a relative measure compared to background uptake by standardizing the duration and starting time for SUV measurements across scans.

Overall, we observed that the duration of the dynamic [^18^F]NaF PET scan can be reduced while maintaining low errors in bone perfusion (*K*_1_) and metabolism (*K*_i_) estimation from the Hawkins model. Shorter dynamic [^18^F]NaF PET scans, potentially as short as 30 min, could be used without appreciable error in Hawkins uptake parameters in bone regions with both low and high tracer uptake.

## Data Availability

The original contributions presented in the study are included in the article/**Supplementary Material**, further inquiries can be directed to the corresponding author.
