# Supplementary material for: Effects of dynamic [18F]NaF PET scan duration on kinetic uptake parameters in the knee
Source: Front Nucl Med. 2023 Nov 24;3:1194961. doi: 10.3389/fnume.2023.1194961 (PMC11440834; doi:10.3389/fnume.2023.1194961)
Supplement: Supplementary file 1 [file Datasheet1.docx]

**Supplemental Material**

**Supplemental Table 1**: Mean and standard deviation of bone region volume

| **Bone** | **Bone type** | **Region** | **Volume (mL)** |
| --- | --- | --- | --- |
| Patella | Cortical |  | 5.41 ± 2.01 |
| Tibia | Cortical | Medial | 8.45 ± 3.38 |
|  |  | Lateral | 8.55 ± 3.15 |
|  |  | Shaft | 8.61 ± 3.95 |
|  | Trabecular | Medial | 16.11 ± 7.46 |
|  |  | Lateral | 23.60 ± 8.62 |
| Femur | Cortical | Medial Trochlea | 1.94 ± 1.01 |
|  |  | Lateral Trochlea | 1.52 ± 0.82 |
|  |  | Medial Central | 5.50 ± 2.30 |
|  |  | Lateral Central | 3.73 ± 1.47 |
|  |  | Medial posterior | 7.99 ± 4.47 |
|  |  | Lateral Posterior | 6.33 ± 2.31 |
|  |  | Shaft | 17.80 ± 5.11 |
|  | Trabecular | Medial | 31.64 ± 11.96 |
|  |  | Lateral | 29.29 ± 9.63 |

**Supplemental Table 2**: Mean and standard deviation of the﻿ y-axis intercept of the Patlak plot (V_0_)

| Time | V_0_ |
| --- | --- |
| 50 | 0.0595 ± 0.1064 |
| 45 | 0.0564 ± 0.0976 |
| 40 | 0.0531 ± 0.0926 |
| 35 | 0.0489 ± 0.0838 |
| 30 | 0.0445 ± 0.0779 |
| 25 | 0.0409 ± 0.0691 |
| 20 | 0.0403 ± 0.0668 |

**Supplemental Figure 1**: Example of an IDIF from one subject plotted together with activity from venous blood samples. Venous blood samples at 50 min matched concentration values from the IDIF at that time point with an average deviation of 0.2 kBq/mL
